# Saccharin Increases Fasting Blood Glucose but Not Liver Insulin Resistance in Comparison to a High Fructose-Fed Rat Model

**DOI:** 10.3390/nu10030341

**Published:** 2018-03-12

**Authors:** Avshalom Leibowitz, Ariel Bier, Mayan Gilboa, Edna Peleg, Iris Barshack, Ehud Grossman

**Affiliations:** 1 Internal Medicine D, The Chaim Sheba Medical Center, Tel-Hashomer, Ramat-Gan 5265601, Israel; mayangilboa@yahoo.com (M.G.); Ehud.Grossman@sheba.health.gov.il (E.G.); 2 Hypertension Unit, The Chaim Sheba Medical Center, Tel-Hashomer, Ramat-Gan 5265601, Israel; arielbier@gmail.com (A.B.); Edna.Peleg@sheba.health.gov.il (E.P.); 3 Sackler Faculty of Medicine, Tel-Aviv University, Tel-Aviv 69978, Israel; Iris.Barshack@sheba.health.gov.il; 4 Institute of Pathology, The Chaim Sheba Medical Center, Tel-Hashomer, Ramat-Gan 5265601, Israel

**Keywords:** fatty liver, fructose, insulin resistance, metabolic syndrome, saccharin

## Abstract

Recent data indicate that artificial sweeteners (AS) may have deleterious effects on glucose metabolism. The purpose of this study was to compare the effects of AS and the effects of a high fructose diet (HFrD) on glucose metabolism and insulin resistance (IR) in Sprague-Dawley (SD) rats. SD rats were fed either regular chow, chow with saccharin (Sac) (0.1 mg/mL) placed in their water, or HFrD for seven weeks. Glucose, insulin, and triglycerides (Tg) levels were measured upon completion. A homeostatic model assessment (HOMA)-IR index was used to determine insulin resistance. The liver was stained to detect signs of a fatty liver. Hepatic mRNA expression of glucose metabolism regulation genes, Srepb-1c (sterol regulatory element binding protein) and ChREB (α & β) (carbohydrate response element binding protein), as well as other glycolytic and lipogenic genes including glucose-6-phosphatase (G6pc), were considered IR markers. Both HFrD and Sac significantly increased fasting blood glucose levels compare to the control (140 ± 5 and 137 ± 6 vs. 118 ± 3 mg/dL, respectively, *p* < 0.05). However, only HFrD increased insulin secretion (0.99 ± 0.12 vs. 0.7 ± 0.1 and 0.6 ± 0.1 ug/L), Tg levels (420 ± 43 vs. 152 ± 20 and 127 ± 13 mg/dL), and the HOMA-IR index (3.4 ± 0.4 vs. 2.3 ± 0.36 and 2.13 ± 0.3) (HFrD vs. control and sac, *p* < 0.05). Fatty liver changes were only observed in HFrD fed rats. The expression of ChREB β, Srepb-1c, and G6pc mRNA were only significantly elevated (between 2–10 times folds, *p* < 0.05) in HFrD fed rats. Sac may increase fasting blood glucose but has no effect on liver insulin resistance.

## 1. Introduction

The prevalence of obesity has steadily increased since the 1980s [1]. Consequently, there has been a significant increase in the prevalence of metabolic syndrome (MeS) and diabetes mellitus (DM), which explains why cardiovascular disease (CVD) has remained the leading cause of mortality in the USA [2] and in other developed and developing countries [3,4]. One explanation for the increase in obesity prevalence relates to the extensive consumption of the “western diet” [1]. This diet is no longer limited only to eating habits of the western world, but is also liberally used in the developing world due to the low cost of processed foods. The “western diet” is characterized by high calories, high fats, high salt, and high fructose content. A high fructose diet can lead not only to obesity, but also to other conditions linked to MeS that are considered as major CVD risk factors, i.e., systemic hypertension (HTN), insulin resistance, and a fatty liver. A fatty liver or “nonalcoholic fatty liver disease” (NAFLD) is a leading cause of liver disease in the modern world. Fat accumulation in the liver, once considered a benign condition, is presently well-recognized as the beginning of a pathological process that may lead to end-stage liver disease and cirrhosis [5]. NAFLD highly correlates with CVD [6]. The presence of NAFLD in imagining studies is a good predictor for the development of CVD [7]. The CVD outcome of patients with NAFLD is poor, including a higher mortality rate [8]. Several features of NAFLD are also common components of MeS, hence, the presence of NAFLD actually indicates the presence of MeS [9], which may explain the significance of an NAFLD-associated CVD risk. Fructose consumption has tripled worldwide during the past few decades; therefore, the impact of fructose on CVD is very significant [10].

Due to the demand to sweeten foods with a low calorie content, artificial sweeteners (AS) were developed and have become very popular. AS were recommended as a sugar substitute for diabetic patients and an important component of weight reduction plans. The metabolic benefits of AS are ambiguous and evidence from clinical trials is controversial. Several studies have demonstrated the metabolic benefits of AS, but others have failed to substantiate any advantages. Some studies have shown detrimental effects such as weight gain and the development of insulin resistance [11]. A recent comprehensive review claimed that AS consumption is associated with high risks of weight gain, heart disease, diabetes, high blood pressure, and obesity [12]. Recently, Suez et al. found that AS may change the microbiota and increase blood glucose levels [13], leading many clinicians to believe that AS have the same detrimental effect on metabolic parameters as fructose and should therefore be avoided. Thus, the aim of the present study was to compare the metabolic effects of AS versus fructose in Sprague-Dawley (SD) male rats.

## 2. Materials and Methods

### 2.1. Animals

Sprague-Dawley (SD) male rats, weighing 200 ± 20 g, were obtained from the Harlan Laboratories, Jerusalem, Israel. The rats were housed in regular cages situated in an animal room at 22 °C using a 14-h light (6:00–20:00)/10-h dark (20:00–6:00) cycle and free access to food and drink. All procedures performed were in accordance with the Chaim Sheba Medical Center guidelines for animal studies and approved by the Institutional Animal Ethics Committee.

### 2.2. Study Protocol

For seven weeks, 30 SD male rats were fed either regular chow (*n* = 10; control (Ctrl)), regular chow with saccharin (Sac) 0.1 mg/mL placed in their drinking water (*n* = 10; Sac), or a high fructose diet (*n* = 10; HFrD). Body weight (BW) and blood pressure (BP) were measured at baseline and upon completion of the study. Fasting blood glucose, insulin, and triglycerides (Tg) levels were also measured on completion. The HOMA2-IR index was used to determine insulin resistance. Upon conclusion, the rats were anesthetized with 3% isoflurane (depth of anesthesia confirmed by rear foot squeezing) and their organs harvested. Tissues were weighed and tibia length determined for standardization. Several portions of liver tissue were quickly removed, snap-frozen in liquid N_2_, and stored at −80 °C. Frozen tissues were used for RNA extraction.

### 2.3. Diets

The standard chow diet (Teklad 2018S) consisted of 44.2% carbohydrates, 18.6% protein, 6.2% fat, 18.2% fibers (crude and natural detergent), 5.3% ash, and a standard vitamin and mineral mix. The HFrD (TD.89247) consisted of 60% fructose, 21% protein, 5% fat, and a standard vitamin and mineral mix (Casein 207, Fructose 600, Lard 50, Cellulose 80, Mineral Mix 50, Vitamin Mix 10, g/kg) (Teklad diets, Envigo, Madison, WI, USA).

#### Metabolic Parameters

Glucose and Tg levels were assayed with an automated analyzer for an enzymatic colorimetric reaction (Olympus AU 2700, Hamburg, Germany). An insulin ELISA kit (10-1250-01, Mercodia AB, Uppsala, Sweden) was used to measure insulin levels. The HOMA-2 IR index was calculated by a free online calculator (HOMA Calculator, Version 2.2.3, Diabetes Trail Unit, The University of Oxford, Oxford, UK). Systolic BP was measured by the indirect tail cuff method, using an electrosphygmomanometer and pneumatic pulse transducer (58500 BP Recorder, UGO BASILE, Varese, Italy). The mean of five consecutive readings determined systolic BP.

### 2.4. Liver Histological Studies

A liver tissue slice fixed in 4% paraformaldehyde was embedded in paraffin for histological studies. Sections from the paraffin embedded liver were stained with hematoxylin and eosin for steatosis assessment. A validated scoring system was used for the quantification of fatty liver changes (Table 1) [14]. Evaluations were performed by a pathologist blinded to the treatment groups. Images were captured by an Olympus BX50 microscope equipped with a digital camera (Olympus DP71) (Olympus Europa SE & CO. KG, Hamburg, Germany).

### 2.5. Real-Time Quantitative Reverse Transcription PCR

The mRNA expression levels of gene involvement in carbohydrate metabolism in the liver (glucose-6-phosphatase, catalytic subunit (G6pc), sterol regulatory element binding transcription factor 1 (Srebp1c)), carbohydrate-responsive element-binding protein α (ChREBPα, also known as MLXIPL), carbohydrate-responsive element-binding protein β (ChREBPβ), pyruvate carboxylase (Pc), glycogen phosphorylase L (Pygl), and phosphoenolpyruvate carboxykinase 1 (Pck1)), were ascertained in the liver tissue by real-time quantitative reverse transcriptase (RT) PCR (qRT-PCR). Total RNA was extracted from the liver tissue by the NucleoSpin RNA Kit (MACHEREY-NAGEL, Düren, Germany). Reverse transcription was performed using the Applied Biosystems High Capacity cDNA Reverse Transcription Kit (Applied Biosystems, Foster City, CA, USA). qRT-PCR reactions were performed by the Power Sybr Green PCR Master Mix (Applied Biosystems, Warrington, UK) using the Applied Biosystems 7500 real-time PCR system. Ribosomal protein lateral stalk subunit P0 (Rplp0) mRNA was used as an internal control. The primers are listed in Table 2.

### 2.6. Statistical Analysis

Data are presented as mean ± S.E.M. Statistical analysis was performed using the IBM^®^ SPSS^®^ Statistics, version 24 (IBM Corporation, Armonk, NY, USA). One-way analysis of variance (ANOVA) and the post hoc Tukey method examined the differences between groups. Real-time qPCR data were analyzed using DataAssist software, version v3.01 (Applied Biosystems, Life Technologies Corporation 2012, now under Thermo Fisher Scientific, Waltham, MA, USA). A *p* value of ≤0.05 was considered statistically significant.

## 3. Results

### 3.1. The Effects of Saccharin Versus a High Fructose Diet on Metabolic Parameters

Body weight increased to the same extent in all groups (Ctrl-408 ± 9, Sac-407 ± 9 and HFrD-415 ± 8 g). Rats fed HFrD developed “metabolic-like” syndrome as expressed by the development of HTN and elevated levels of Tg (Table 3). In addition, these rats demonstrated impaired glucose metabolism including high fasting blood glucose, high insulin levels, and a high HOMA-IR index. Rats fed with Sac exhibited only high fasting blood glucose but no other features of metabolic-like syndrome (Table 3).

### 3.2. The Effects of Saccharin Versus a High Fructose Diet on Fatty Changes in the Liver

**Figure 1:**
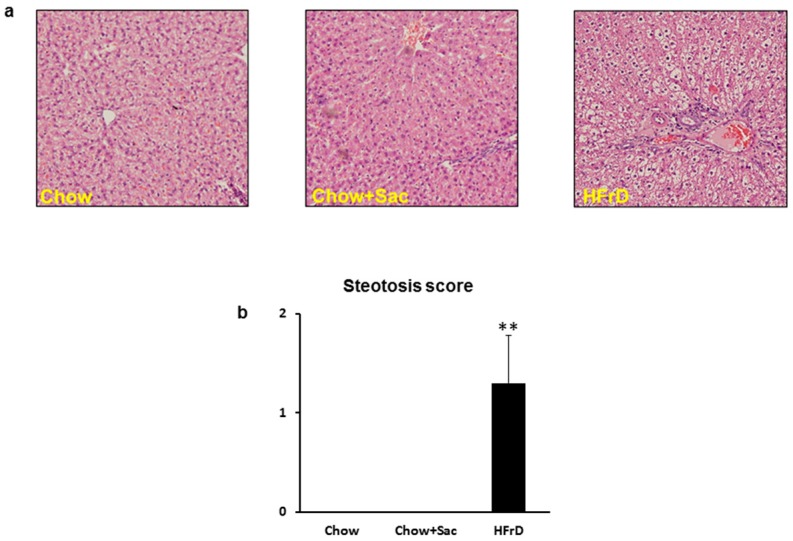
Rats fed HFrD developed a fatty liver. (**a**) Representative slides, hematoxylin eosin staining of liver tissue showing steatosis changes only in rats fed HFrD (high fructose diet) but not in the Ctrl (control) or Sac (saccharine) group (40× magnification, *n* = 9–10); (**b**) steatosis score-ranked by a pathologist according to an objective scale. ** *p* < 0.01 vs. chow and chow + Sac.

Rats fed HFrD exhibited heavier livers than the Ctrl and Sac groups (Ctrl-3.8 ± 0.08 and Sac-3.8 ± 0.08 vs. HFrD-4.5 ± 0.08, *p* < 0.05, corrected to BW). The histological appearance of the HFrD-fed rats’ livers, showed clear micro-vesicular steatosis changes not observed in the livers of the Ctrl and Sac groups (Figure 1).

### 3.3. The Effects of Saccharin Versus a High Fructose Diet on the Expression of Genes Linked to Insulin Resistance in the Liver

**Figure 2:**
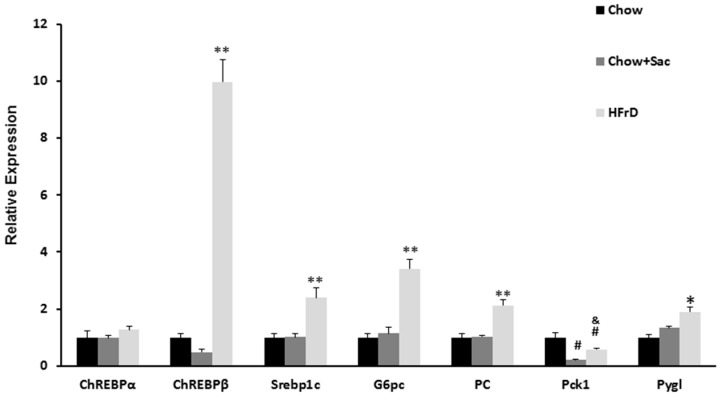
Fructose, not saccharin, increased the expression of genes in the liver which regulates CH metabolism, liver DNL, gluconeogenesis, and glycogenolysis. ChREBPα-carbohydrate-responsive element-binding protein α, ChREBPβ-carbohydrate-responsive element-binding protein β, Srebp1c-sterol regulatory element binding transcription factor 1, G6pc-glucose-6-phosphatase, catalytic subunit, PC-pyruvate carboxylase, Pck1-phosphoenolpyruvate carboxykinase 1, Pygl-glycogen phosphorylase. # *p* < 0.05 vs. chow; * *p* < 0.05 vs. chow and chow + Sac; ** *p* < 0.01 vs. chow and chow + Sac; & *p* < 0.05 vs. Sac.

We studied the expression of several genes associated with carbohydrate metabolism of the liver. No changes were observed in the expression of ChREBPα BP mRNA, a pivotal transcriptional regulator of glycolytic and lipogenic genes. However, HFrD induced a significant increase in the expression of its isoform ChREBPβ, a more potent gene modulator. Sac had no effect on the expression of this isoform. The expression of ChREBPβ is considered a marker for ChREBP activity with high activity indicating insulin resistance. Similarly, HFrD, not saccharin, increased the expression of Srebp1c, a transcriptional regulator of hepatic de novo lipogenesis (DNL). The patterns of gluconeogenic genes were not consistent, as the levels of G6pc and Pc, but not Pck1, were elevated in the HFrD group. The glycogen cleavage enzyme Pygl expression expected to be low when blood glucose was elevated, was significantly higher only in the HFrD group (Figure 2).

## 4. Discussion

We compared the metabolic effects of Sac and fructose, both used as commercial sweeteners, in an animal model and found that Sac increased fasting blood glucose in exactly the same manner as fructose. However, unlike fructose, Sac produced no negative effects on other metabolic parameters such as Tg, insulin, and the HOMA index. Moreover, HFrD, induced histological fatty liver changes, whereas Sac had no detrimental effect on the liver. The histological findings were in accordance with the in vitro results as only HFrD increased the expression of several genes involved in carbohydrate metabolism (indicating the presence of insulin resistance), whereas Sac produced no such effect.

Our data suggest that Sac definitely increases blood glucose, but does not cause metabolic-like syndrome. The data with regard to the effect of AS in humans is intriguing. In a nine-year follow-up study of dietary habits, Lutsey et al. found that the consumption of diet soda was associated with MeS [15]. In Nettleton et al.’s cohort study, a daily consumption of diet soda was found to be associated with a greater risk of MeS and type 2 DM compared with non-consumption. The authors did not compare the effect of diet soda with sugar consumption [16]. In another cohort study, de Koning et al. reported that while the consumption of sugar sweetened beverages was associated with a significantly elevated risk of type 2 diabetes, the association between artificially sweetened beverages and type 2 diabetes was largely due to baseline health status and body weight of the individual rather than by AS use [17]. In contrast to these findings, Tate et al. observed that the replacement of caloric beverages with non-caloric beverages resulted in significant weight loss [18]. Human observational studies have yielded limited insight into possible mechanisms; hence, animal studies are warranted.

However, evidence from animal models is also not consistent. Amin et al. reported that Sac decreased body weight, fasting blood glucose, total and LDL-cholesterol, and Tg levels in Rattus Norvegicus albino rats, but increased liver transaminases (TANs) compared to a control group fed normal chow [19]. Alkafafy et al. compared the consumption of Sac versus a normal diet without Sac in Wistar albino rats and found that Sac decreased body weight and increased TANs levels [20]. Several other studies noted body weight reduction and several changes in biochemical liver function tests [21,22]; however, none of these studies compared Sac to fructose. Since Sac is an alternative sweetener, our study compared the metabolic effects of fructose and Sac. We evaluated the effects of the different diets on liver histology and gene expression, but did not measure TANs, since as previously reported, TANs were not elevated in this model of HFrD fed rats [23]. Suez et al. showed that AS, including Sac, may increase blood glucose in the gut microbiota of mice. The authors failed to indicate changes in insulin secretion, raising the question as to whether AS induces insulin resistance [13]. In concordance with their findings, we determined that Sac increases fasting blood glucose with no effect on insulin secretion. Several measures were used to compare the effects of Sac and fructose on metabolic syndrome. In all parameters, fructose induced insulin resistance and MeS, while Sac did not.

In a study investigating the role of endotoxin in the pathogenesis of fructose-induced fatty liver, Bergheim et al. used Sac sweetened water as a control treatment. In accordance with our findings, they reported that Sac increased fasting blood glucose without any effect on body weight or TANs. Moreover, similar to our findings, the authors observed that sugar-fed mice accumulated lipids in the liver, whilst Sac-fed mice did not [24].

As was mentioned in the introduction, NAFLD is a major CVD risk factor and its presence as part of the MeS may predict an adverse cardiovascular sequel. Our important finding in the present study is that despite a high glucose level, Sac-fed rats did not develop a fatty liver. Hence, high glucose levels are not sufficient enough to diagnose MeS as there is no correlation with tissue damage. The presence of insulin resistance is crucial for the development of NAFLD, even in lean subjects [25].

Fructose consumption is a well-known NAFLD “inducer”. According to the “two hit” theory, fructose contributes to liver steatosis by promoting insulin resistance and liver de novo lipogenesis during the “first hit” and triggering inflammatory and oxidative stress processes during the “second” [26]. The exact mechanism of liver insulin resistance is as yet not fully understood. A common explanation is the presence of “selective insulin resistance” where only the arm regulating gluconeogenesis is impaired, while DNL remains intact or even enhanced [27]. Another theory postulates that hepatic insulin resistance is mediated by other metabolic pathways independent of insulin-receptor signaling. Herman et al. recently described a new potent isoform of ChREBP-ChREBP β, which is associated with carbohydrate metabolism and insulin resistance [28]. They demonstrated that ChREBP β regulated carbohydrate metabolism in a fructose-fed rat model [29]. In accordance with the above results, we report that a fructose-rich diet, without Sac, induced liver up-regulation of the same genes mentioned in Herman et al.’ study, i.e., ChREBP β, G6pc, and others. Our results indicate that despite elevating fasting plasma glucose such as fructose, Sac does not metabolically affect the liver.

### Study Limitations

Our study has some limitations. Due to technical issues, the treatments were delivered to the rats in a different manner; fructose in the diet and Sac in the drinking water. Therefore, one should be cautious when translating the animal data to real life. Second, we focused on the ChREBP pathway in this study, ignoring insulin signaling pathways. However, recent studies have emphasized the ChREBP pathway in the regulation of fructose metabolism in the liver. Third, unfortunately, we have no data on pancreas gene expression nor on c-peptide level.

## 5. Conclusions

In conclusion, Sac increases fasting blood glucose, but has no adverse metabolic effects on the liver’s carbohydrate metabolism. Hence, we can suggest that it is safe to consume Sac, even for patients with obesity or diabetes, as its consumption does not lead to the development of NAFLD and MeS.

## Figures and Tables

**Table 1:** Scoring system used for quantification of fatty liver changes.

**Steatosis parenchymal involvement**	<5%	0
	5–33%	1
	33–66%	2
	>66%	3
**Location**	Zone 3	0
	Zone 1	1
	Azonal	2
	Panacinar	3
**Microvesicular steatosis**	Not present	0
	Present	1
**Fibrosis**	None	0
	Perisinusoidal/periportal	1
	Perisinusoidal and periportal	2
	Bridging fibrosis	3
	Cirrhosis	4

**Table 2:** Oligonucleotides used in qRT-PCR assays.

Gene Name	Forward	Reverse
Rplp0 *	GAACATCTCCCCCTTCTCCTTC	ATTGCGGACACCCTCTAGGAA
PC	CCAAGCAGGTTGGCTATGAGAA	GATGTTTTCCTGCCGCAGCC
Pck1	GGATGTGGCCAGGATCGAAA	ATACATGGTGCGGCCTTTCA
ChREBP-α	TGCATCGATCACAGGTCATT	AGGCTCAAGCATTCGAAGAG
ChREBP-β	TCTGCAGATCGCGCGGAG	CTTGTCCCGGCATAGCAAC
G6pc	CGTCACCTGTGAGACTGGAC	ACGACATTCAAGCACCGGAA
Pygl	ATAATTGGTGGGAAAGCTGCC	GCCAGCAGTGGAGATCTGTT
Srebp-1c	CATGGATTGCACATTTGAAGAC	GCAGGAGAAGAGAAGCTCTCAG

* The ribosomal protein lateral stalk subunit P0 (Rplp0) gene was chosen as a housekeeping molecule for relative quantification.

**Table 3:** Rats’ body weight, blood pressure, and triglyceride levels while on the various diets **.

Type Diet	Ctrl	Saccharin	HFrD
Body weight (g)	408 ± 9	407 ± 9	415 ± 8
Blood pressure (mmHg)	135 ± 1	138 ± 1	156 ± 2 *
Triglycerides (mg/dL)	152 ± 20	127 ± 13	420 ± 43 *
Glucose (mg/dL)	118 ± 3	137 ± 6 #	140 ± 5 ^#^
Insulin (ug/L)	0.7 ± 0.1	0.6 ± 0.1	0.99 ± 0.12 *
HOMA2-IR	2.3 ± 0.36	2.13 ± 0.3	3.4 ± 0.4 *

Ctrl, control; HFrD, high fructose diet; Sac, saccharine. * *p* < 0.05 vs. Ctrl & Sac, ^#^ *p* < 0.05 vs. chow. ** measures were taken after seven weeks of diet administration.

## References

[1] The GBD 2015 Obesity Collaborators (2017). Health Effects of Overweight and Obesity in 195 Countries over 25 Years. N. Engl. J. Med..

[2] Benjamin E.J., Blaha M.J., Chiuve S.E., Cushman M., Das S.R., Deo R., de Ferranti S.D., Floyd J., Fornage M., Gillespie C. (2017). Heart Disease and Stroke Statistics-2017 Update: A Report from the American Heart Association. Circulation.

[3] Prabhakaran D., Roy A., Praveen P.A., Ramakrishnan L., Gupta R., Amarchand R., Kondal D., Singh K., Sharma M., Shukla D.K. (2017). 20-Year Trend of cardiovascular disease risk factors: Urban and rural national capital region of Delhi, India. Glob. Heart.

[4] Heidenreich P.A., Trogdon J.G., Khavjou O.A., Butler J., Dracup K., Ezekowitz M.D., Finkelstein E.A., Hong Y., Johnston S.C., Khera A. (2011). Forecasting the future of cardiovascular disease in the United States: A policy statement from the American Heart Association. Circulation.

[5] Peter M., Tommy N. (2017). Non-alcoholic fatty liver disease-a chronic disease of the 21st century. J. Biomed. Res..

[6] Ekstedt M., Hagstrom H., Nasr P., Fredrikson M., Stal P., Kechagias S., Hultcrantz R. (2015). Fibrosis stage is the strongest predictor for disease-specific mortality in NAFLD after up to 33 years of follow-up. Hepatology.

[7] Arulanandan A., Ang B., Bettencourt R., Hooker J., Behling C., Lin G.Y., Valasek M.A., Ix J.H., Schnabl B., Sirlin C.B. (2015). Association between quantity of liver fat and cardiovascular risk in patients with nonalcoholic fatty liver disease independent of nonalcoholic steatohepatitis. Clin. Gastroenterol. Hepatol..

[8] Stepanova M., Rafiq N., Makhlouf H., Agrawal R., Kaur I., Younoszai Z., Valasek M.A., Ix J.H., Schnabl B., Sirlin C.B. (2013). Predictors of all-cause mortality and liver-related mortality in patients with non-alcoholic fatty liver disease (NAFLD). Dig. Dis. Sci..

[9] Kitade H., Chen G., Ni Y., Ota T. (2017). Nonalcoholic fatty liver disease and insulin resistance: New insights and potential new treatments. Nutrients.

[10] Lustig R.H., Schmidt L.A., Brindis C.D. (2012). Public health: The toxic truth about sugar. Nature.

[11] Swithers S.E. (2013). Artificial sweeteners produce the counterintuitive effect of inducing metabolic derangements. Trends Endocrinol. Metab..

[12] Azad M.B., Abou-Setta A.M., Chauhan B.F., Rabbani R., Lys J., Copstein L., Mann A., Jeyaraman M.M., Reid A.E., Fiander M. (2017). Nonnutritive sweeteners and cardiometabolic health: A systematic review and meta-analysis of randomized controlled trials and prospective cohort studies. Can. Med. Assoc. J..

[13] Suez J., Korem T., Zeevi D., Zilberman-Schapira G., Thaiss C.A., Maza O., Israeli D., Zmora N., Gilad S., Weinberger A. (2014). Artificial sweeteners induce glucose intolerance by altering the gut microbiota. Nature.

[14] Kamari Y., Shaish A., Vax E., Shemesh S., Kandel-Kfir M., Arbel Y., Olteanu S., Barshack I., Dotan S., Voronov E. (2011). Lack of interleukin-1alpha or interleukin-1beta inhibits transformation of steatosis to steatohepatitis and liver fibrosis in hypercholesterolemic mice. J. Hepatol..

[15] Lutsey P.L., Steffen L.M., Stevens J. (2008). Dietary intake and the development of the metabolic syndrome: The Atherosclerosis Risk in Communities study. Circulation.

[16] Nettleton J.A., Lutsey P.L., Wang Y., Lima J.A., Michos E.D., Jacobs D.R. (2009). Diet soda intake and risk of incident metabolic syndrome and type 2 diabetes in the Multi-Ethnic Study of Atherosclerosis (MESA). Diabetes Care.

[17] De Koning L., Malik V.S., Rimm E.B., Willett W.C., Hu F.B. (2011). Sugar-sweetened and artificially sweetened beverage consumption and risk of type 2 diabetes in men. Am. J. Clin. Nutr..

[18] Tate D.F., Turner-McGrievy G., Lyons E., Stevens J., Erickson K., Polzien K., Diamond M., Wang X., Popkin B. (2012). Replacing caloric beverages with water or diet beverages for weight loss in adults: Main results of the Choose Healthy Options Consciously Everyday (CHOICE) randomized clinical trial. Am. J. Clin. Nutr..

[19] Amin K.A., AlMuzafar H.M. (2015). Alterations in lipid profile, oxidative stress and hepatic function in rat fed with saccharin and methyl-salicylates. Int. J. Clin. Exp. Med..

[20] Alkafafy Mel S., Ibrahim Z.S., Ahmed M.M., El-Shazly S.A. (2015). Impact of aspartame and saccharin on the rat liver: Biochemical, molecular, and histological approach. Int. J. Immunopathol. Pharmacol..

[21] Andrejic B.M., Mijatovic V.M., Samojlik I.N., Horvat O.J., Calasan J.D., Dolai M.A. (2013). The influence of chronic intake of saccharin on rat hepatic and pancreatic function and morphology: Gender differences. Bosn. J. Basic Med. Sci..

[22] Parlee S.D., Simon B.R., Scheller E.L., Alejandro E.U., Learman B.S., Krishnan V., Bernal-Mizrachi E., MacDougald O.A. (2014). Administration of saccharin to neonatal mice influences body composition of adult males and reduces body weight of females. Endocrinology.

[23] Ackerman Z., Oron-Herman M., Grozovski M., Rosenthal T., Pappo O., Link G., Sela B.A. (2005). Fructose-induced fatty liver disease: Hepatic effects of blood pressure and plasma triglyceride reduction. Hypertension.

[24] Bergheim I., Weber S., Vos M., Kramer S., Volynets V., Kaserouni S., McClain C.J., Bischoff S.C. (2008). Antibiotics protect against fructose-induced hepatic lipid accumulation in mice: Role of endotoxin. J. Hepatol..

[25] Marchesini G., Brizi M., Morselli-Labate A.M., Bianchi G., Bugianesi E., McCullough A.J., Forlani G., Melchionda N. (1999). Association of nonalcoholic fatty liver disease with insulin resistance. Am. J. Med..

[26] Lim J.S., Mietus-Snyder M., Valente A., Schwarz J.M., Lustig R.H. (2010). The role of fructose in the pathogenesis of NAFLD and the metabolic syndrome. Nat. Rev. Gastroenterol. Hepatol..

[27] Brown M.S., Goldstein J.L. (2008). Selective versus total insulin resistance: A pathogenic paradox. Cell Metab..

[28] Herman M.A., Peroni O.D., Villoria J., Schon M.R., Abumrad N.A., Bluher M., Klein S., Kahn B.B. (2012). A novel ChREBP isoform in adipose tissue regulates systemic glucose metabolism. Nature.

[29] Kim M.S., Krawczyk S.A., Doridot L., Fowler A.J., Wang J.X., Trauger S.A., Noh H.L., Kang H.J., Meissen J.K., Blatnik M. (2016). ChREBP regulates fructose-induced glucose production independently of insulin signaling. J. Clin. Investig..

